# Real-Time Monitoring of Jet Trajectory during Jetting Based on Near-Field Computer Vision

**DOI:** 10.3390/s19030690

**Published:** 2019-02-08

**Authors:** Jinsong Zhu, Wei Li, Da Lin, Ge Zhao

**Affiliations:** 1 School of Mechatronic Engineering, China University of Mining and Technology, Xuzhou 221116, China; cmeezjs@cumt.edu.cn (J.Z.); linda_cmee@cumt.edu.cn (D.L.); zero166@126.com (G.Z.); 2 Xu-gong Construction Machinery Group (XCMG) Research Institute, Xuzhou 221116, China

**Keywords:** automatic fire suppression systems, jet trajectory, real-time monitoring, near-field computer vision

## Abstract

A novel method of near-field computer vision (NFCV) was developed to monitor the jet trajectory during the jetting process, which was used to precisely predict the falling point position of the jet trajectory. By means of a high-resolution webcam, the NFCV sensor device collected near-field images of the jet trajectory. Preprocessing of collected images was carried out, which included squint image correction, noise elimination, and jet trajectory extraction. The features of the jet trajectory in the processed image were extracted, including: start-point slope (SPS), end-point slope (EPS), and overall trajectory slope (OTS) based on the proposed mean position method. A multiple regression jet trajectory range prediction model was established based on these trajectory characteristics and the reliability of the model was verified. The results show that the accuracy of the prediction model is not less than 94% and the processing time is less than 0.88 s, which satisfy the requirements of real-time online jet trajectory monitoring.

## 1. Introduction

With public safety requirements becoming increasingly important in various countries, fire safety equipment is being developed in terms of automation and intelligence [1,2]. More and more urban complexes are required to have automatic fire suppression systems installed, and various types of fire-fighting vehicles are also required to continuously improve their automation level [3,4,5]. Therefore, firewater reaching the point of fire quickly and accurately has grown to be an area of interest for researchers. During the fire-fighting process, identification and location of the fire source and automatic control of the jet trajectory falling position are the most crucial parts of the automatic fire suppression system [6,7,8]. Currently, there are a lot of investigations into the identification and location of fire sources, while few studies on jet trajectory have been carried out. In most cases, the efficiency of fire water reaching the fire position largely depends on the empirical model. However, the process of fire-fighting is very complicated, these areas being full of smoke, toxic gas, high temperatures, and the possibility of explosion. The challenge not only involves extinguishing the flames accurately but also ensuring the safety of the firefighters. Therefore, real-time monitoring of the jet trajectory not only improves the automation of the fire-fighting process, but also greatly reduces the time required for fire protection. Furthermore, real-time monitoring of the jet trajectory is extraordinary necessary to make the fire water arrive as soon as possible after the fire source is located. For the past few years, computer vision has commonly been used in many fields, such as fire detection [9,10,11,12,13], the mineral separation industry [14,15], intelligent measurements [16,17,18], and the processing of agricultural products [19,20,21]. With the aid of high-speed networks and advanced processing algorithms, computer vision has shown automation, accuracy, and intelligence advantages in the field of industrial process control. In this study, jet trajectory measurement technology based on near-field computer vision is investigated to predict the falling position of a water jet.

In recent years, computer vision technology has become an important direction in the development of automatic fire suppression systems because it can improve extinguishment efficiency and precision. For example, McNeil [22] proposed an autonomous fire suppression system including a multispectral sensor suite to suppress wood crib fires in indoor environments with high or low visibility. Kim [23] developed a real-time probabilistic classification method for identifying fire, smoke, their thermal reflections, and other objects in infrared images. Foggia [9] proposed a method that is able to detect fires by analyzing videos acquired by surveillance cameras. Turgay [10] proposed a rule-based generic color model for flame pixel classification and the method achieves up to a 99% fire detection rate. Additionally, an IR vision system which is capable of identifying water based on a real-time probabilistic classification method was presented [24]. In this method, a single IR camera is utilized for collecting the images and acquiring textural features of high-motion regions calculated with a three-frame difference approach. Furthermore, McNeil applied a computer vision-based autonomous fire suppression system with real-time feedback of fire size and water jet direction [25]. In order to predict the path water discharge trajectory with a large capacity monitor, Miyashita [26] established a jet trajectory prediction model by fitting a third order function to experimental data based on flow rate and pressure. However, computer vision technology in automatic fire suppression systems is still in its preliminary stage and is limited in practical application due to the harsh environment in the fire-fighting process. Because of this harsh, it is very difficult for the image acquisition device to capture a complete water jet trajectory [27,28,29]. Furthermore, taking into account the working conditions of automatic fire suppression systems and the processing of the jet trajectory detection, there are still some key technical issues waiting to be resolved, such as the development of complete water jet image acquisition technology, high-speed image processing, prediction of the water jet trajectory falling position, and so on. These issues restrict the application of computer vision in automatic fire suppression systems. Therefore, a new jet trajectory detection method is required for automatic fire suppression systems that is real time, high accuracy, reliable, and can work within a harsh environment in the fire-fighting process.

As mentioned in the previous paragraph, automatic fire suppression systems need the water jet to reach the fire location precisely. In the process of fire-fighting, the range of water jet trajectories is so far that it is difficult for the image acquisition device to capture the full jet trajectory. Therefore, near-field computer vision can be applied. Near-field computer vision means that part of the jet trajectory image is used instead of the whole trajectory image to predict the falling position. The falling position of a jet trajectory is mainly determined by two factors: the initial jet angle and its velocity. Previous studies have established various types of jet trajectory models based on the angle and velocity, such as the experiment-based jet trajectory model [30,31] and simulation-based jet trajectory model [32]. So far, the established trajectory models provide values of the initial angle and velocity calculated from the location of fire source relative to fire cannon. As a result, the falling position of the jet trajectory can be given while adjusting the initial angle and velocity. However, manual intervention is still required if the trajectory placement position has to be adjusted. Therefore, real-time monitoring of jet trajectories based on computer vision is necessary while it is still difficult to capture a complete water jet trajectory.

The objective of our work is to develop a method for real-time monitoring of the jet trajectory during jetting. In order to overcome the difficulty of capturing the complete trajectory of the jet, a near field trajectory image is used instead. Integrating the characteristics of the near-field trajectory image, we propose a novel jet trajectory detection method based on visible light; the proposed detection method is named near-field computer vision (NFCV). It is a new method to detect the jet trajectory in this area. In the NFCV method, near the fire monitor, part of the jet trajectory image is obtained by the proposed NFCV sensor device, which is based on the visible camera and the image conversion algorithm interactive interface. In the NFCV method, three features of near-field water jet trajectory images are extracted for the trajectory placement prediction. The use of the proposed NFCV method makes jet trajectory detection and falling position control possible in the complex fire-fighting environment. The remainder of the paper is organized as follows: Section 2 presents the NFCV method. Section 3 provides the experiment and analysis to verify the reliability and accuracy of the NFCV method. Section 4 gives the overall conclusion.

The major contributions of the manuscript can be summarized as follows:

(1) Implementing a fully real-time NFCV-based system that is capable of generating a multiple regression jet trajectory range prediction model with corresponding feature extraction.

(2) A real-time image processing method for segmentation and extraction of jet trajectory features is introduced, and the processing time is less than 0.88 s.

(3) Accurate prediction of falling point, the results showing that the prediction error of falling point position is less than 6%.

## 2. The NFCV Method

The NFCV method consists of four major parts, near-field image capture, image preprocessing, jet trajectory feature extraction, and jet trajectory model. Firstly, the NFCV sensor device is used to capture the jet trajectory image. Second, the perspective transformation and image preprocessing algorithms are used to process the image, so as to better extract the jet trajectory feature. Finally, the trajectory feature is extracted by the detection algorithm. All parts will be described in a more detailed way in the following sections.

### 2.1. Near-Field Image Capture

**Figure 1:**
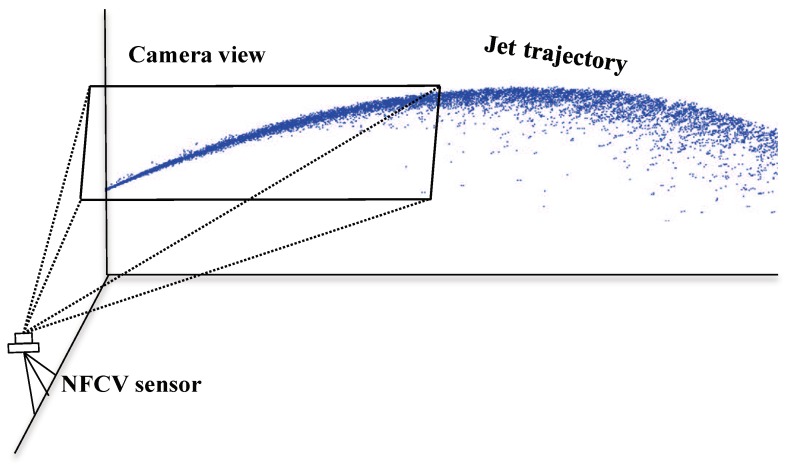
The structure of the near-field computer vision (NFCV) sensor device.

It is necessary to capture high-quality images in vision detection for jet trajectory. The NFCV vision sensor device is designed to capture the first-stage trajectory image while it is difficult to capture the trajectory image completely during jetting. The NFCV vision sensor device includes two parts: the vision sensor device and the computer processing interface. The vision sensor device is composed of a visible camera and a bracket device, the visible camera being fixed on the bracket device. In the process of fire-fighting, the falling position of the water jet is far away from the fire monitor, this makes it difficult to capture a complete jet trajectory image at the location of the fire monitor. Simultaneously, it is also unrealistic to capture the trajectory image with the vision sensor device away from the fire monitor. Therefore, the visible camera is installed 40 cm away from the fire cannon, which makes it possible to shoot the jet trajectory from an oblique angle. The structure of the NFCV vision sensor device is shown in Figure 1.

As shown in Figure 1, the jet trajectory images are captured by the visible camera continuously during jetting. The visible camera acts as an image acquisition tool which inputs the live preview image of the jet trajectory to the graphical user interface (GUI) developed in MATLAB. Each video frame captured by the visible camera has a resolution of 1280 × 960 pixels in the RGB format.

### 2.2. Image Preprocessing

#### 2.2.1. Perspective Transformation

After a near-field image is acquired, the original image must be restored to the front view through perspective transformation. The range of jet trajectories is fairly large, thus it is difficult to obtain its full image. Therefore, the jet trajectory image acquired by the near-field sensor device must be processed by the perspective transformation algorithm. Perspective transformation [33,34] is the projection of an image onto a new viewing plane (front view plane), the transition function can be written as

(1) $$\left[x^{\prime },y^{\prime },z^{\prime }\right]=\left[u,v,w\right]*\left[\begin{array}{ccc} a_{11} & a_{12} & a_{13}\\ a_{21} & a_{22} & a_{23}\\ a_{31} & a_{32} & a_{33} \end{array}\right]$$

where $\left[u,v\right]$ are the coordinates of the original image pixel and $\left[x=\frac{x^{\prime }}{w^{\prime }},y=\frac{y^{\prime }}{w^{\prime }}\right]$ is the pixel coordinates of the image after transformation. A further explanation is as follows:

- The linear transformation matrix:
  (2) $$T_{1}=\left[\begin{array}{cc} a_{11} & a_{12}\\ a_{21} & a_{22} \end{array}\right].$$
- The perspective transformation matrix:
  (3) $${\left[\begin{array}{cc} a_{13} & a_{23} \end{array}\right]}^{T}.$$
- The translation matrix:
  (4) $$\left[\begin{array}{cc} a_{31} & a_{32} \end{array}\right].$$

**Figure 2:**
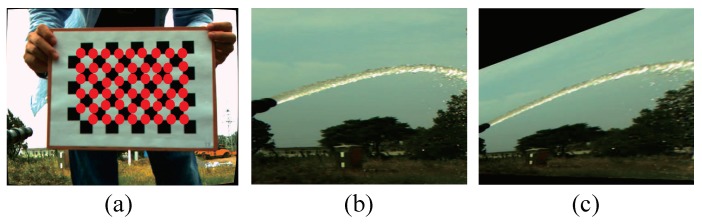
The method of perspective transformation for: (**a**) Corner detection; (**b**) jet trajectory before perspective transformation; (**c**) jet trajectory after perspective transformation.

In our work, the perspective transformation matrix is obtained by detecting the corner of the calibration plate. The angular point detection of the calibration plate is shown in Figure 2, and the perspective matrix can be expressed as

(5) $$\left[\begin{array}{ccc} 2.0731 & -0.1143 & -87.4407\\ -0.7044 & 1.0061 & 54.0602\\ -0.0001 & 0.0001 & 1 \end{array}\right].$$

#### 2.2.2. Image Enhancement

**Figure 3:**
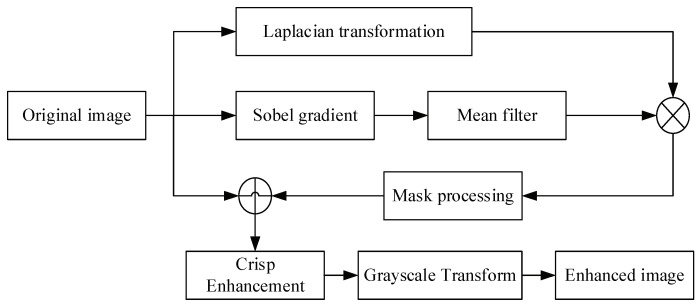
The image enhancement flow diagram of the jet trajectory original image.

In the process of image acquisition, noise will be generated because of the poor working environment of fire-fighting. That is disadvantageous for extracting image features. Jet trajectories are generally white or bright in the image, as water tends to reflect light more easily. Therefore, enhancement of the gray value of the jet trajectory in the image is the key to image enhancement. In this paper, the improved cooperative algorithm [35,36] is used to enhance and eliminate noise. The process of the image enhancement algorithm is shown in Figure 3.

**Figure 4:**
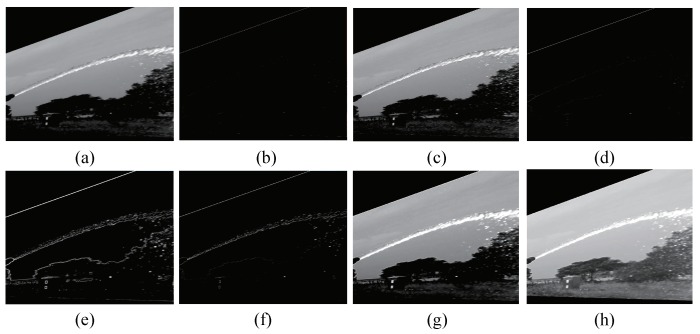
Example of calculation results for each step in the image enhancement process. (**a**) Original image; (**b**) Laplacian transformation; (**c**) a + b; (**d**) Sobel gradient; (**e**) mean filter; (**f**) masking image; (**g**) crisp enhancement; (**h**) grayscale transform.

The steps to perform a cooperative image enhancement can be summarized as follows:

(1) The Laplacian transformation enhances the contrast of the gray mutation in the image and the details of the image which are becoming smaller and smaller, whilst preserving the background tone of the image, making the details of the image clearer than the original image.

(2) Add the original image to Figure 4b, a sharpened image can be obtained.

(3) The Sobel gradient is used to detect pixels with step changes in the gray level around the jet trajectory, and the set of these pixels is the edge of the trajectory.

(4) A 5 × 5 mean filter is used to smooth the image of the Sobel gradient.

(5) Figure 4c multiplies Figure 4e to realize the mask processing of the image, and some parts of the image are screened out.

(6) Add the Figure 4f to Figure 4b to obtain a further sharpened image.

(7) Perform grayscale conversion on Figure 4g obtained in step six to extend the gray scale range of the image.

With the improved cooperative enhancement algorithm, Figure 4 shows the result of each step, it can be observed that the enhanced image is sharper than the original image. Furthermore, the jet trajectory is brighter than other regions in the image. In other words, the enhanced image is more convenient for the segmentation of the jet trajectory.

#### 2.2.3. Image Segmentation

In the enhanced image, there are obvious differences between the jet trajectory and background. Universally, the gray value of the background is lower than that of the jet trajectory. Before introducing the segmentation approach, we describe a series of events that can be observed as changes in the gray histogram before and after the image enhancement.

**Figure 5:**
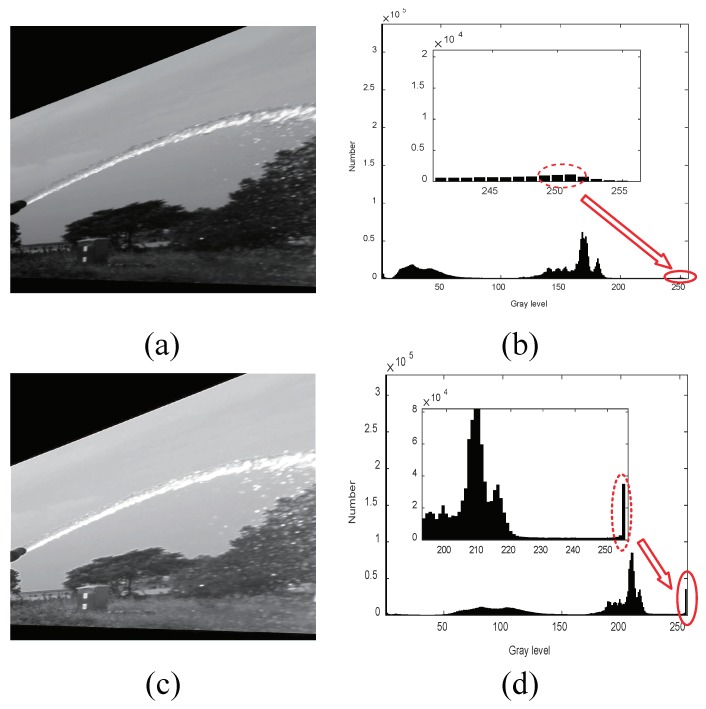
Results image with the proposed image enhancement method. (**a**) Original image; (**b**) gray histogram of (**a**); (**c**) enhanced image; and (**d**) Gray histogram of (**b**).

As shown in Figure 5a, the black shadow, ground, and sky are the areas where the image gray value changes greatly. As a result, the black shadow, ground, and sky in the original image produce a new mode in the histogram with a peak, with a low gray value and high gray value agglomeration area, respectively. Although the gray value of the jet trajectory is relatively high in the original image, it is still submerged in the high gray value region of the gray histogram, and after the improved cooperative image enhancement processing, it is as shown in Figure 5c. In the enhanced image, the jet trajectory changes more obviously. This resulted in a new small peak at the extreme right side of the gray histogram, as shown in the red circle in Figure 5d, it was considered as an enhanced jet trajectory.

**Figure 6:**
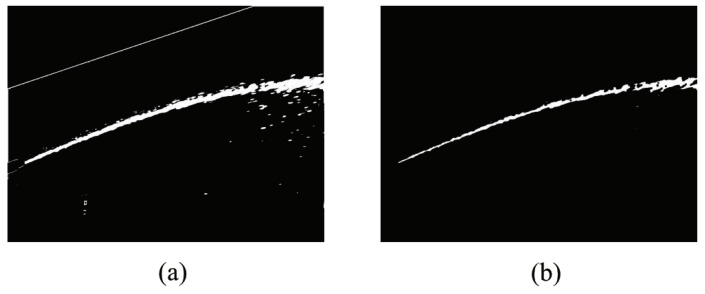
Results image with morphological treatment. **(a)** Original image; **(b)** morphologically processed image.

Since the jet trajectory is separated from the high gray value region in the original gray histogram, a new peak is formed on the right side of the enhanced gray histogram. Therefore, the new peak gray value can be used to easily identify the jet trajectory effectively through the segmentation approach. In order to determine the segmentation gray threshold value accurately, the new peak value in 100 enhanced images is selected and the results show the new peak always fluctuates between 230 and 255. In order to assure the accuracy of segmentation, the threshold should be chosen carefully. In our work, the length threshold is set to 230. The results of Figure 5a after segmentation are as shown in Figure 6a.

#### 2.2.4. Morphological Operation

After image enhancement and segmentation, the jet trajectory is extracted accurately. However, there is still some noise, which is mainly from the bright small areas in the original image. Morphological operations are used to eliminate the noise in Figure 6, such as the falling water drop, distribution cabinets, and the slender curves produced by perspective transformation. In our work, the mathematical morphology [37,38,39] is utilized to eliminate the interference. On the basis of mathematical morphology, the structure element selected a disk of 5 × 5. The opening operation is expressed as below:

- Dilation:
  (6) $$X⊕B=\left\{p\in \varepsilon^{2}:p=a+b,x\in X,b\in B\right\}.$$
- Erosion:
  (7) $$X\Theta B=\left\{p\in \varepsilon^{2}:p+b\in X,\forall b\in B\right\}.$$
- Opening operation:
  (8) $$X\circ B=\left(X\Theta B\right)⊕B.$$

Where *X* denotes the image, B is the structure element. In the segmentation result shown in Figure 6, the morphologically processed jet trajectory image becomes cleaner, and falling water droplets and other environmental disturbances are eliminated.

### 2.3. Jet Trajectory Feature Extraction

This part includes trajectory curve fitting and feature extraction. In this paper, we propose a detection algorithm, called the mean position method, to extract the jet trajectory coordinates and fit the trajectory curve.

#### 2.3.1. Mean Position Method

**Figure 7:**
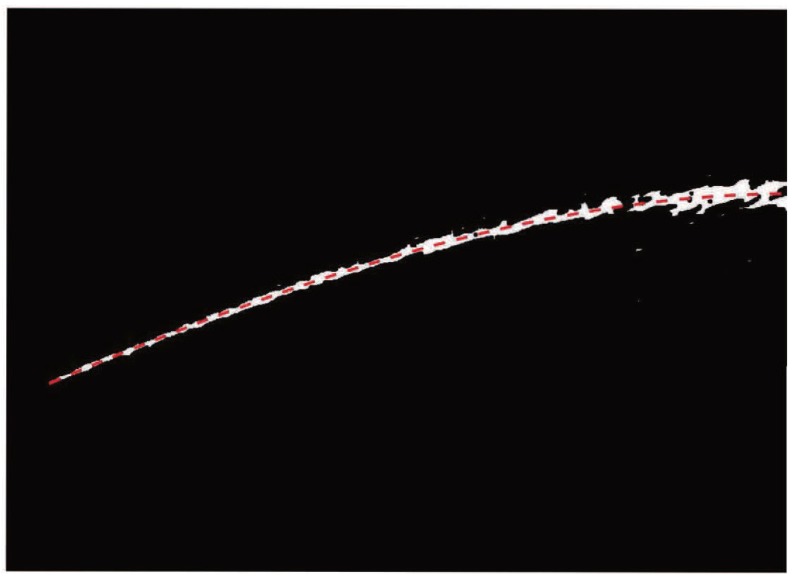
The jet trajectory curve based on the proposed mean position method.

As we know, the jet trajectory position parameters in the binary image are the data foundation for establishing the trajectory equation. However, in the binary image, the thickness of the jet trajectory is different and there is more than one pixel in the longitudinal direction of the image [40,41]. Furthermore, there is still noise in the pre-processed jet trajectory image, which could affect the establishment of the jet trajectory equation. In order to obtain the position of the jet trajectory in the image, a novel approach is proposed based on the mean position method. Suppose the size of the binary image $g\left(i,j\right)$ is m×n, the ordinate of the jet trajectory in the binary image based on the developed mean position method are expressed as

(9) $$Y\left(i\right)=\frac{1}{a}\sum_{j=1}^{n}(n-j)\;g\left(i,j\right)=1$$

where *a* is the number of pixels that satisfies $g\left(i,j\right)=1$. Y(i) is the ordinate of the jet trajectory in the column of the binary image. The mean value of the vertical coordinate of each column of jet path pixels in the binary image is selected based on the proposed method, which is considered to be the ordinate of the jet trajectory curve in that column. Figure 7 shows the jet trajectory curve drawn based on the calculated coordinate data under Figure 6b. By comparing the jet trajectory drawn with those in the binary graphs under the same coordinate system, it is easy to find that the trajectory curve is basically in the middle of the jet trajectory in the binary image and they basically overlap with each other.

#### 2.3.2. Feature Extraction

On the basis of the proposed mean position method, the coordinates of the jet trajectory in the binary images are obtained, and the trajectory curve equation is fitted by means of the fitting toolbox. In our work, three features are proposed to characterize the range of trajectory, including: start-point slope (SPS), end-point slope (EPS), and overall trajectory slope (OTS). The detailed introduction is described below:

(10) $$SPS=\frac{dy}{dx}\left(x_{1}\right)$$

(11) $$EPS=\frac{dy}{dx}\left(x_{n}\right)$$

(12) $$OTS=\frac{y\left(x_{n}\right)-y\left(x_{0}\right)}{x_{n}-x_{0}}$$

where y(x) is the trajectory curve equation, $x_{0}$ and $x_{n}$ are the starting and ending point abscissa of the jet trajectory curve equation, respectively. The physical meaning of each feature is explained as follows:

SPS is the starting point slope of the jet trajectory equation, which represents the initial angle of the jet trajectory. EPS is the slope of jet trajectory equation at the end point of abscissa of the binary image, which represents the motion direction of the jet trajectory while it breaks away from the camera view. OTS is the slope value of the beginning and end of jet trajectory in the binary image, which represents the motion state of the jet trajectory in the entire camera view. As illustrated in Figure 7, the SPS, EPS, and OTS of the jet trajectory in the binary image coordinate system are 0.46, 0.01, and 0.25, respectively. Furthermore, when SPS = 0.44, the initial angle of the jet trajectory is 24.75°. It should be noted that the result of experiment shows that the detected and calculated initial jet angles are identical and the feature representation is feasible.

The NFCV method is used to obtain the initial part jet trajectory image, and clean initial jet trajectory binary images are obtained through transforming, enhancing, and segmenting the image. The mean position method is used to obtain the coordinates of the jet trajectory in the binary image and the trajectory curve equation is fitted based on the coordinate position data in the same coordinate system as the binary image.

## 3. Experimental Results and Discussions

In order to verify the proposed NFCV method, an experimental facility was constructed in the Xu-gong Construction Machinery Group (XCMG) Comprehensive Test Site, and experiment results were analyzed to prove the reliability of the proposed characterization methods. The experimental system and experiment results analysis are introduced as follows.

### 3.1. Experiment Setup

**Figure 8:**
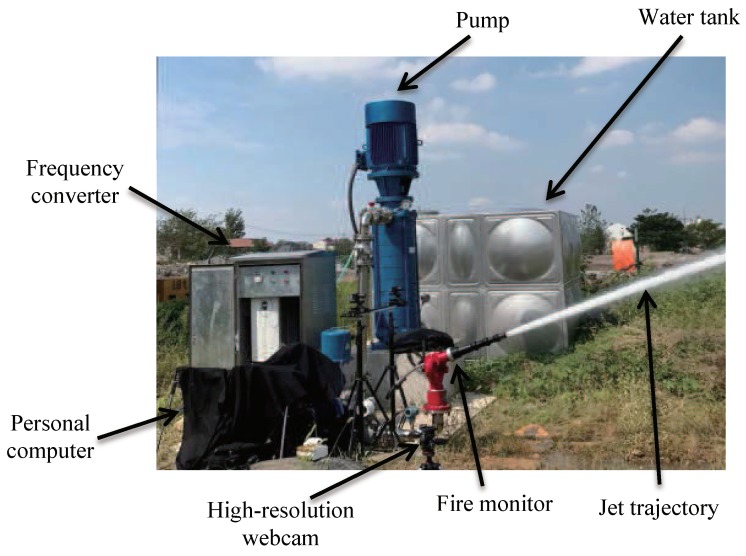
The experiment platform of the NFCV method.

The experimental system was mainly composed of two parts: the water jet equipment and the NFCV sensor device, as shown in Figure 8. The experimental platform consisted of a water tank, pump, frequency converter, personal computer, high-resolution webcam, fire monitor, and other fittings. Furthermore, the water tank provided 10 tons of water in one experiment, and the water was pressurized into the pipeline through a pump controlled by the frequency converter. In our experimental system, the direct current fire monitor PS20-50 was selected, this being one of the most commonly used fire monitors. The exit diameter of the experimental fire monitor was 33 mm and the water entering the monitor was controlled by the pump. In other words, the fire monitor output speed of the water was determined by the frequency of the frequency converter. In our work, the outlet velocity of water jet was approximately linear with the pump frequency. Pump frequencies of 10, 20, 30, and 40 Hz corresponded to the outlet velocities of 5.4, 14.0, 22.1, and 31.1 m/s, respectively. The proposed NFCV device consisted of a lens, a CCD camera, a tripod, and a computer, their detailed configuration is described in Section 2.1.

The proposed NFCV device acquires the original jet trajectory image with the image resolution of 1280 × 960 with a rate of 6 frames per second. Moreover, the MATLAB software is used to process images and extract features. At the same time, the GUI is designed to display the trajectory image and the predicted jet trajectory falling position. The experiment mainly includes the following steps:

(1) The initial discharge angle of the fire monitor is set to $10^{\circ }$, and the frequency range of the converter is set to be from 5 Hz to 40 Hz.

(2) Open the pump of the water jet experimental system. By means of the NFCV device, the image of the jet trajectory is obtained at 6 frames per second and the range of the trajectory is recorded, respectively.

(3) Set the initial discharge angle of the fire monitor to $15^{\circ }$, $20^{\circ }$, $25^{\circ }$, $30^{\circ }$, and $35^{\circ }$, respectively, repeat the two steps mentioned above.

**Figure 9:**
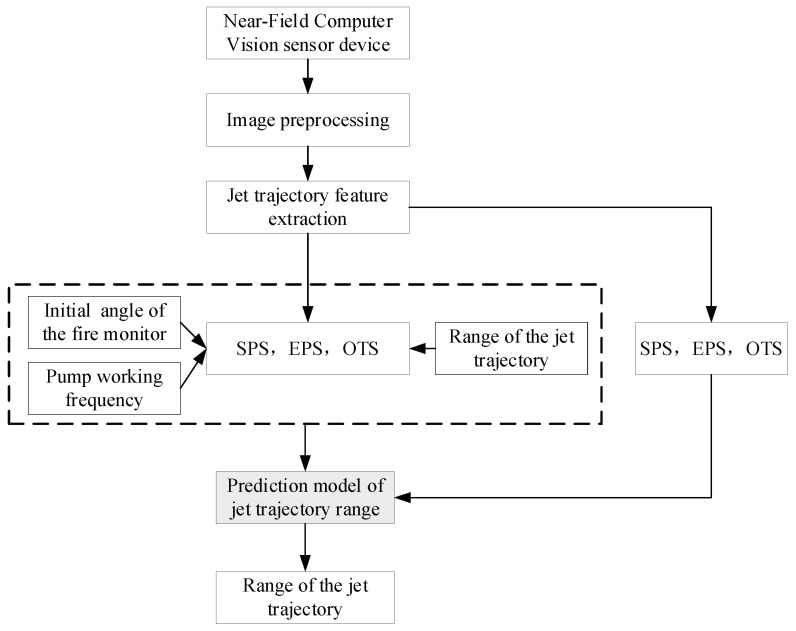
The experiment platform of the NFCV method.

The experiment is done six times and 180 sets of original image are obtained in each experiment. The flowchart of the NFCV method is shown in Figure 9. The experimental results and analysis are shown in the following section.

### 3.2. Experiment Results and Analysis

**Figure 10:**
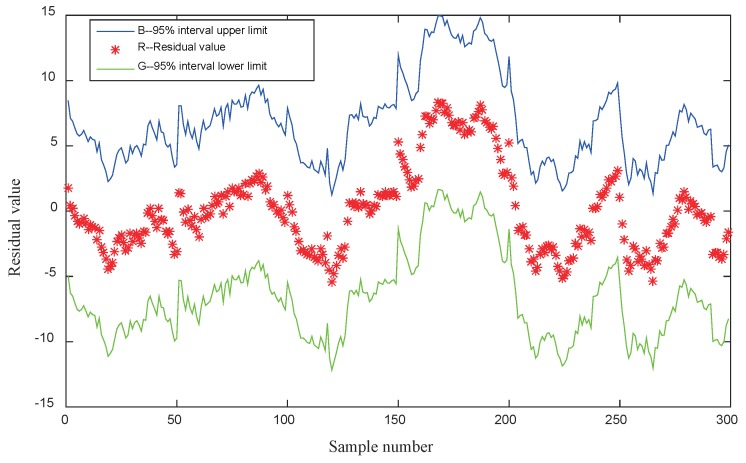
Residual graph of the multiple regression models.

Three characteristics of jet trajectories were extracted by means of the NFCV method, the actual jet trajectories range was also measured and recorded. In our work, 150 sets of data including SPS, EPS, OTS, and the actual range of the jet trajectory were obtained for prediction model creation, and the other 30 sets of data were used for model validation. With the aid of the least square method, a multivariate regression model for the prediction of the jet trajectory range was established based on collected data. The basic principle of the prediction model can be expressed as

(13) $$y=\beta_{0}+\beta_{1}x_{1}+\beta_{2}x_{2}+\beta_{3}x_{3}$$

where $\beta_{0}$ is the regression constant, $\beta_{1},\beta_{2},\beta_{3}$ are the regression coefficients, $x_{1}$, $x_{2}$, and $x_{3}$ correspond to the three trajectory features of SPS, EPS, and OTS, respectively. The judgment coefficient of the prediction model can be expressed as

(14) $$R2=\frac{SSR}{SST}=1-\frac{SSE}{SST}=1-\frac{\sum {\left(y-\overset{\wedge }{y}\right)}^{2}}{\sum {\left(y-\bar{y}\right)}^{2}}$$

where SSR, SSE, and SST are the sum of the regression squares, the sum of residuals squares, and the sum of total deviation squares, respectively. In this paper, the regression constant is 27.3691 and the regression coefficients are 0.2895, 0.2126, 0.2833, and the judgment coefficient of the prediction model is 0.94, which indicates that the prediction model is highly fitted. As shown in Figure 10, the residual values of the regression model are all within the confidence interval, which indicates that the regression model is in line with the requirements.

**Figure 11:**
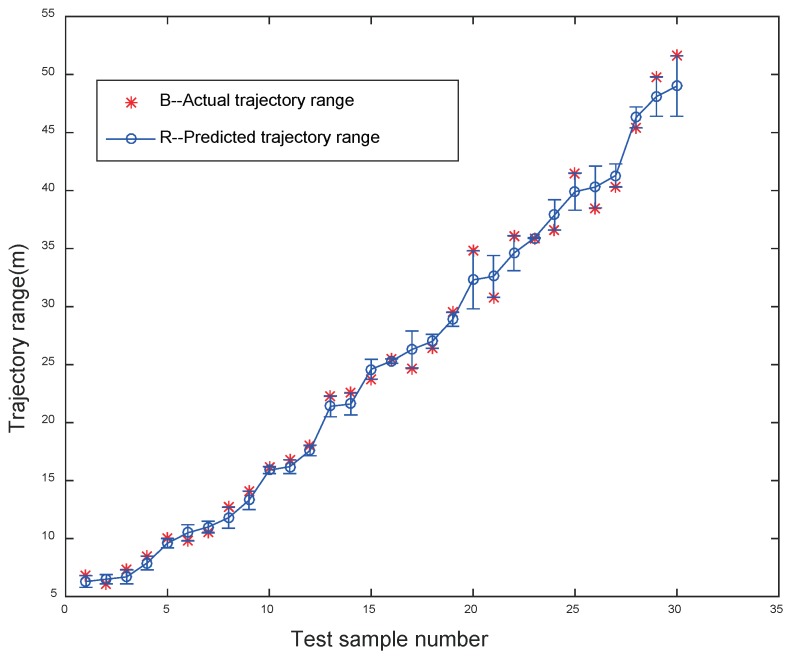
Residual graph of the multiple regression models (red and blue dots represent the predicted and actual values of the trajectory range, respectively).

To verify the reliability of the trajectory prediction model, 30 sets of unused data were used to test the regression model, and the results are shown in Table 1, Table 2, and Figure 11.

The predicted results of the jet trajectory range are shown in Table 1. There are 20 groups with an error range of less than 1 m, 67% of the total. Their average error is 0.59 m and the average percentage error is 4.35%. It can be seen from the results that the average error is 0.59 m and the average error percentage is 4.35%. There are eight errors ranging from 1.0 m to 2.0 m, and the average error value and the average error percentage are 1.54 m and 4.23%, respectively. Furthermore, only errors of two predicted jet trajectory ranges are greater than 2 m, and the average error value and the average error percentage are 2.55 m and 6.52%, respectively. Moreover, the average processing time of each prediction of the jet trajectory range is no more than 0.88 s.

Meanwhile, the superiority of the proposed method is further demonstrated in Table 2 by comparison with the jet trajectory prediction spreadsheet model proposed by Miyashita. As can be seen from Table 2, the NFCV method shows the advantages in the accuracy of falling position prediction of the jet trajectory. Furthermore, all the prediction errors of the NFCV method are less than 10%, and most of them are less than 5%. Small and stable error results are important to guide the operation of the automatic fire extinguishing system. More importantly, the processing time of the NFCV method is less than 0.88 s, which makes it feasible for real-time trajectory location analysis and prediction. This advantage is not available in other methods while the timeliness of this method allows the jet trajectory to be adjusted in real time during the fire-fighting process.

It can be seen from Figure 11 that the prediction error increases with the increase in the actual jet trajectory range. The maximum prediction error of 2.6 m occurs when the actual range of jet trajectory is 49 m, which is the maximum range of the jet trajectory measured in our experiment. Moreover, the prediction error from 1.0 m to 2.0 m also mostly occurs in the actual jet trajectory range when greater than 30 m. Correspondingly, the prediction error of less than 1 m mostly occurs when the actual jet trajectory range is less than 30 m. The phenomenon of the prediction error results from the open experiment environment, in which the disturbance of the jet trajectory range by the breeze increases as the range increases.

## 4. Conclusions

In this paper, a novel NFCV method is proposed for real-time monitoring of the jet trajectory during jetting by computer vision. The initial image of the jet trajectory is acquired with the NFCV sensor device, and the preprocessing of the original image, including perspective transformation, image enhancement, and image segmentation, is carried out to achieve the extraction of the jet trajectory in the acquired image. A novel mean position approach is developed to characterize jet trajectories in binary images and three jet trajectory characteristics (SPS, EPS, and OTS) are extracted to represent the state of the jet trajectory. Moreover, a multivariate regression model for the prediction of the jet trajectory range is established based on the least square method. Experiment results demonstrate that the prediction accuracy of the jet trajectory range is over 94%. For an acquired original image, the NFCV method takes less than 0.88 s on average to finish the jet trajectory range prediction on a personal computer with 3.5 GHz CPU and 16 GB RAM. In summary, the NFCV method meets the requirement of real-time monitoring of jet trajectory during jetting and can be used to further enhance the automation degree of automatic fire suppression systems.

## Figures and Tables

**Table 1:** Analysis of the prediction results of the jet trajectory range.

Error Range (m)	Amount	Average Magnitude of Error(m)	Mean Absolute Percentage Error (%)	Average Processing Time (s)
<1.0	20	0.59	4.35	0.86
1.0–2.0	8	1.54	4.23	0.85
>2.0	2	2.55	6.52	0.88
Mean value	30	0.97	4.46	0.86

**Table 2:** Comparison of two falling position prediction methods of the jet trajectory.

Category	Percentage Error (%)	Amount	Mean Absolute Percentage Error (%)	Average Processing Time (s)
Spreadsheet	<5.0 5.0–10.0 >2.0 Mean value	6 5 1 12	3.6 8.6 14.7 6.3	None
NFCV	<5.0 5.0–10.0 >2.0 Mean value	19 11 0 30	3.1 6.9 0 4.5	0.88 0.87 0 0.88

## References

[1] China Fire Protection Association (2018). Statistical report of the 17th international fire equipment and technology exchange exhibition. Fire Sci. Technol..

[2] Casey Grant (2015). Research Roadmap for Smart Fire Fighting Summary Report. Nat. Inst. Stand. Technol..

[3] Peng R., Zhu B. (2008). Application of Firetrace Automatic Fire Suppression Systems. Build. Sci..

[4] Hopkins M., Schoenrock J., Budnick E.K. (2005). Fire Suppression Systems.

[5] Artim N. (2003). Automatic Fire Suppression for Historic Structures: Options and Applications. APT Bull..

[6] Choi Y.K., Yoon B.D., Kim E.K., Shin M.C. (2011). Development of automatic extinguisher using ignition sensing tube for smart fire protection system. Int. J. Precis. Eng. Manuf..

[7] Kumar K., Sen N., Azid S., Mehta U. (2017). A Fuzzy Decision in Smart Fire and Home Security System. Procedia Comput. Sci..

[8] Mahmud M.S., Islam M.S., Rahman M.A. (2017). Smart Fire Detection System with Early Notifications Using Machine Learning. Int. J. Comput. Intell. Syst..

[9] Foggia P., Saggese A., Vento M. (2015). Real-time Fire Detection for Video Surveillance Applications using a Combination of Experts based on Color, Shape and Motion. IEEE Trans. Circuits Syst. Video Technol..

[10] Celik T., Demirel H. (2009). Fire detection in video sequences using a generic color model. Fire Saf. J..

[11] Zhang Z., Shen T., Zou J. (2014). An Improved Probabilistic Approach for Fire Detection in Videos. Fire Technol..

[12] Toulouse T., Rossi L., Akhloufi M.A., Pieri A., Maldague X. (2018). A multimodal 3D framework for fire characteristics estimation. Meas. Sci. Technol..

[13] Borges P.V.K., Izquierdo E. (2010). A probabilistic approach for vision-based fire detection in videos. IEEE Trans. Circuits Syst. Video Technol..

[14] Qiao T., Chen L., Pang Y., Yan G., Miao C. (2017). Integrative binocular vision detection method based on infrared and visible light fusion for conveyor belts longitudinal tear. Measurement.

[15] Yu B., Qiao T., Zhang H., Yan G. (2018). Dual band infrared detection method based on mid-infrared and long infrared vision for conveyor belts longitudinal tear. Measurement.

[16] Che J.K., Ratnam M.M. (2018). Real-time monitoring of workpiece diameter during turning by vision method. Measurement.

[17] Leal-Muñoz E., Diez E., Perez H., Vizan A. (2018). Accuracy of a new online method for measuring machining parameters in milling. Measurement.

[18] Chen Z., Zhang Z., Dai F., Bu Y., Wang H. (2017). Monocular vision-based underwater object detection. Sensors.

[19] Daniel P., Diego G., García J. (2015). Sorting olive batches for the milling process using image processing. Sensors.

[20] Wang W., Xin B., Deng N., Li J., Liu N. (2018). Single vision based identification of yarn hairiness using adaptive threshold and image enhancement method. Measurement.

[21] Dema M., Turner C., Sari-Sarraf H., Hequet E. (2016). Machine Vision System for Characterizing Horizontal Wicking and Drying Using an Infrared Camera. IEEE Trans. Ind. Inform..

[22] McNeil J.G., Lattimer B.Y. (2017). Robotic Fire Suppression Through Autonomous Feedback Control. Fire Technol..

[23] Kim J.H., Lattimer B.Y. (2015). Real-time probabilistic classification of fire and smoke using thermal imagery for intelligent firefighting robot. Fire Saf. J..

[24] McNeil J.G., Lattimer B.Y. (2015). Real-Time Classification of Water Spray and Leaks for Robotic Firefighting. IGI Global.

[25] McNeil J.G., Lattimer B.Y. (2016). Autonomous Fire Suppression System for Use in High and Low Visibility Environments by Visual Servoing. Fire Technol..

[26] Miyashita T., Sugawa O., Imamura T. (2014). Modeling and analysis of water discharge trajectory with large capacity monitor. Fire Saf. J..

[27] Chen T., Yuan H., Su G., Fan W. (2004). An automatic fire searching and suppression system for large spaces. Fire Saf. J..

[28] Yuan H.Y. (2002). Fire location and suppression with automatic hydrant in large space. NRIFD Symp.-Sci..

[29] Nam S., Braga A., Kung H., Troup J. (2003). Fire Protection For Non-storage Occupancies With High Ceiling Clearances. Fire Saf. J..

[30] Xin Y., Thumuluru S., Jiang F. (2014). An Experimental Study of Automatic Water Cannon Systems for Fire Protection of Large Open Spaces. Fire Technol..

[31] Xin Y. Experimental Study on Fire Extinguishing Characteristics of Automatic Sprinkler System. Proceedings of the Sixth International Conference on Intelligent Systems Design and Engineering Applications.

[32] Hatton A.P., Leech C.M., Osborne M.J. (1985). Computer simulation of the trajectories of large water jets. Int. J. Heat Fluid Flow.

[33] Alter T.D. (1994). 3-D Pose from 3 Points Using Weak-Perspective. IEEE Trans. Pattern Anal. Mach..

[34] Carlbom I., Paciorek J. (1978). Planar Geometric Projections and Viewing Transformations. ACM Comput. Surv..

[35] Gilboa G., Sochen N., Zeevi Y.Y. (2004). Image enhancement and denoising by complex diffusion processes. IEEE Trans. Pattern Anal. Mach. Intell..

[36] Lee J.S. (2009). Digital image enhancement and noise filtering by use of local statistics. IEEE Trans. Pattern Anal. Mach. Intell..

[37] Zhou X., Podoleanu A.G., Yang Z., Yang T., Zhao H. (2012). Morphological operation-based bi-dimensional empirical mode decomposition for automatic background removal of fringe patterns. Opt. Express.

[38] Hao G., Ono N., Sagayama S. (2008). A Structure-Synthesis Image Inpainting Algorithm Based on Morphological Erosion Operation. Congr. Image Signal Process..

[39] Chudasama D., Patel T., Joshi S., Prajapati G.I. (2015). Image Segmentation using Morphological Operations. Int. J. Comput. Appl. Technol..

[40] Sallam K.A., Dai Z., Faeth G.M. (2002). Liquid breakup at the surface of turbulent round liquid jets in still gases. Int. J. Multiph. Flow.

[41] Zhang W., Zhu D.Z. (2015). Far-field properties of aerated water jets in air. Int. J. Multiph. Flow.

